# The relationship between pregnancy and birth experience with maternal-fetal attachment and mother-child bonding: a descriptive-analytical study

**DOI:** 10.1186/s40359-023-01475-x

**Published:** 2023-12-05

**Authors:** Monireh Moniri, Fatemeh Rashidi, Mojgan Mirghafourvand, Mansour Rezaei, Solmaz Ghanbari-Homaie

**Affiliations:** 1 Student Research Committee, Tabriz University of Medical Sciences, Tabriz, Iran; 2 Social Determinants of Health Research Center, Tabriz University of Medical Sciences, Tabriz, Iran; 3 Department of Anesthesiology, School of Medicine, Tabriz University of Medical Sciences, Tabriz, Iran; 4 Department of Midwifery, Faculty of Nursing and Midwifery, Tabriz University of Medical Sciences, Shariati Street, P.O. Box: 51745-347, 513897977 Tabriz, Iran

**Keywords:** Attachment, Bonding, Postpartum, Pregnancy, Birth satisfaction, Maternal-fetal relations

## Abstract

**Background:**

Pregnancy and childbirth experience can be important factors for a pleasant relationship between mother and baby. This study assessed the relationship between the pregnancy and birth experience with maternal-fetal attachment (MFA) and mother-child bonding.

**Methods:**

A descriptive-analytical study was conducted among 228 pregnant women in Tabriz, Iran February 2022 to March 2023. Using cluster random sampling method, we included 228 women with gestational age 28–36 weeks and followed them up until six weeks postpartum. Data were collected in two stages using the following questionnaires: Pregnancy Experience Scale (hassles and uplifts), Maternal-Fetal Attachment Questionnaire (during the third trimester of pregnancy), Postpartum Bonding Questionnaire, and Childbirth Experience Questionnaire (six weeks postpartum). Data were analyzed using Pearson’s correlation test and general linear model.

**Results:**

The mean score of MFA was significantly higher among women with feelings of being uplifted during pregnancy [β (95% CI) = 1.14 (0.87 to 1.41); p < 0.001]. However, there was no statistically significant relationship between pregnancy hassles and MFA and mother-child bonding (p > 0.05). Also, there was no statistically significant relationship between childbirth experience and mother-child bonding (p > 0.05).

**Conclusion:**

According to the results of this study, pregnancy uplifts have a positive role in improving MFA. Therefore, it is recommended to plan interventions to make pregnancy period a pleasant experience for mothers.

## Background

Maternal-fetal attachment (MFA) is defined as “the degree to which women actively participate in behaviors that reflect a sense of connection and interaction with their unborn child”. It is the manifestation of care and concern towards the fetus, expressed through various means such as affection, emotions, perceptions, concerns, and expectations [1]. The next stage is called mother-child bonding, which means the feelings that the mother experiences about her new baby [2]. It is determined by the mother’s behaviors, including looking, smiling, touching, and talking with the child, which starts growing almost before pregnancy [3].

The mother-child attachment is the most important relationship in every person’s life, which is formed from the embryonic period and affects the entire subsequent stages of a person’s life, such as social, emotional, and cognitive development, as well as future life. Therefore, it is essential to understand the nature of MFA and bonding and their predictors [4]. The satisfactory mother and child bonding is crucial for fostering positive socio-emotional development in children because it can influence various aspects of their personality, such as socialization skills, self-confidence, honesty, and independence [2]. Some factors such as mother’s mental health, social support, marital satisfaction [5, 6], pregnancy experience [6], and fear of childbirth [7] have been proposed as predictors of MFA.

The experience of pregnancy has been proposed as one of the factors related to MFA. The positive experience of the perinatal period is defined as maintaining the optimal physical, social, and cultural condition and the health of the mother and baby during pregnancy, along with performing a safe birth that creates the foundations of a healthy motherhood [8]. The negative experience of pregnancy due to the decrease in sleep quality, physical problems [9], depression, and anxiety during pregnancy might lead to problems in the adjustment of mother and child after birth [4].

Problems related to mental health [10], adaptation to mother’s role [11], kind of birth, and childbirth experience have also been proposed as predictors of mother-child bonding [12]. Childbirth experience is a complex multidimensional and mental experience, which is defined by safe birth and unique physical and cognitive processes experienced by women during labour and birth. One of the positive consequences of childbirth experience is a positive attitude towards motherhood and a safe mother-child bonding [13, 14]. Skin-to-skin contact between mother and baby immediately after birth and early breastfeeding of the baby have a significant effect on creating a positive childbirth experience; it has also been reported that such measurements can strengthen mother-child bonding [12, 15].

There is limited data about the relationship between pregnancy and childbirth experience with MFA and mother-child bonding. To fill this gap, this study aimed to determine the relationship between pregnancy and childbirth experiences with MFA and mother-child bonding.

## Methods

### Study design and participants

This descriptive-analytical study was conducted on 228 pregnant women in Tabriz, Iran from February 2022 to March 2023. All pregnant women (singleton) with gestational age 28–36 weeks were included in the study. The exclusion criteria were as follows: history of cesarean section, giving birth for more than three times, history of depression or postpartum depression, use of anti-depressants, known abnormality in the fetus, and the occurrence of a stressful event such as divorce, death, or diagnosis of an incurable disease for a first-grade family member during the last three months.

### Sample size

The sample size was calculated as 152 women (SD = 5.87, α = 0.05, d = 0.05) around the mean (18.67) of pregnancy experience through n = Z _1−a/2_ sd^2^/d^2^ [16]. Considering the effect size due to the cluster sampling method, the calculated sample size was multiplied by 1.5 (final sample size = 228).

### Sampling

The sampling method was random cluster sampling. Initially, we prepared a comprehensive list of all health centers in Tabriz, Iran (n = 82). Then, we randomly selected a quarter of these centers. The researchers obtained the list of all pregnant women with gestational age 28–36 weeks. Then, the eligible women in each center were selected using simple random sampling. A written informed consent was obtained from all participants prior to the study, and the questionnaires were completed in two stages: [1] during third trimester of pregnancy through face-to-face interview: [sociodemographic and obstetric characteristics questionnaire, The Pregnancy Experience Scale, and Maternal-Fetal Attachment Questionnaire]; [2] six weeks postpartum: [Postpartum Bonding Questionnaire and Childbirth Experience Questionnaire-2]. The researchers followed up the participants once a month by making a phone call. Due to the COVID-19 pandemic and the reluctance of some women to visit in person, mothers who had access to the internet and social networks completed the questionnaires online.

### Data collection tools

To collect data, sociodemographic and obstetric characteristics questionnaire, Pregnancy Experience Questionnaire (PES), Maternal-Fetal Attachment Questionnaire (MFAQ), Childbirth Experience 2.0 (CEQ 2.0), and Postpartum Bonding Questionnaire (PBQ) were used.

### Sociodemographic and obstetric characteristics questionnaire

The sociodemographic and obstetric questionnaire consisted of different items such as age, body mass index (BMI), husband’s age, income status, educational status, husband’s education, job, and husband’s job, as well as obstetrical characteristics, including gestational age, number of pregnancies, number of abortions, pregnancy type (wanted or unwanted pregnancy), type of previous birth, place of birth, satisfaction with pervious birth, having complications during pregnancy, having postpartum complication, having skin to skin contact, anthropometric index of the baby, and baby’s sex. The content validity of this questionnaire was assessed and confirmed by an expert panel, including ten experts in the fields of midwifery, reproductive health, obstetrics, and gynecology.

### The pregnancy experience scale (PES)

The short version of the PES has 20 items with two subscales: hassles (feelings of discomfort) and uplifts (feelings of happiness) during pregnancy. In this scale, a four-point Likert scale (from “not at all” [score 0] to “very much” [score 3]) is assigned to each item. A higher score indicates more discomfort or happiness in hassles and uplifts subscales, respectively. The reliability coefficient of the whole English version has been reported 0.80 [17]. The psychometrics properties of the Persian version were evaluated in Iran by Ebadi et al. Cronbach’s alpha of the whole scale, uplifts, and hassles subscales were reported as 0.71, 0.77, and 0.67, respectively [18].

#### Maternal-fetal attachment questionnaire (MFAQ)

This questionnaire consists of 24 questions divided into five subscales: “interaction with the fetus”, “differentiation of self from fetus”, “role taking”, “attributing character istics to the fetus”, and “giving of self”. Scoring is based on a five-point Likert scale and the options include “yes-always”, “yes-sometimes”, “not sure”, “no-rarely”, and “no-never”. The scoring of all questions is from 1 to 5 and in the case of question 20, it is reversed. A higher score indicates better attachment [19]. The validity and reliability of MFAQ in Iran has been confirmed by Abbasi et al. and Cronbach’s alpha coefficient has been reported as 0.80 [20].

#### Postpartum bonding questionnaire (PBQ)

This questionnaire contains 25 questions and expresses the mother’s feelings and attitude towards the baby. PBQ has four subscales, which subscale 1 reflects impaired bonding (12 items; score range 0 to 60), subscale 2 reflects rejection and anger (7 items; 0 to 35), subscale 3 reflects anxiety about care (4 items; 0 to 20), and subscale 4 is the risk of child abuse (2 items; 0 to 10). The mother expresses her feeling on a 6-point Likert scale (score 0 to 5). Higher scores indicate a more unfavorable relationship [21]. The validation of the Persian version was done by Aflakseir and Jamali, and Cronbach’s alpha coefficient for the components of defective bonding, rejection and anger, care anxiety, and child abuse risk were reported as 0.52, 0.67, 0.70, and 0.74, respectively [22].

### Childbirth experience questionnaire 2.0 (CEQ 2.0)

The CEQ [23] has 23 questions related to four subsacles (“own capacity”, “professional support”, “perceived safety”, and “participation”). The responses include “completely agree” (score 1), “often agree” (score 2), “often disagree” (score 3), and “completely disagree” (score 4). A higher average score indicates more positive experience of childbirth. The validation of the Persian version of CEQ 2.0 has been confirmed by Ghanbari et al. The internal consistency and reliability of the tool have bee reported as 0.93 and 0.97, respectively [24].

### Data analysis

The data were analyzed using the SPSS software, Version 24.0 (IBM Inc., Armonk, NY, USA). We used descriptive statistics for reporting quantitative and qualitative variables, including mean (standard deviation) and frequency (percent). To determine the relationship between pregnancy and birth experience with MFA and mother-child bonding in univariate analysis, Pearson’s correlation coefficient (normal distribution) and Spearman’s correlation coefficient (non-normal distribution) tests were used. In the multivariate analysis, general linear model was used by adjusting socio-demographic and obstetrics characteristics (all variables with p < 0.1 were enterd in the model). A p-value less than 0.05 was considered as statistically significant.

## Results

Out of a total of 228 women included in the study, four participants were excluded due to non-response during the postpartum period. Almost half of the women were primiparous and most of them had no history of abortion or stillbirth. Almost three quarters of women stated that their pregnancy was desired (Table 1).

**Table 1:** Socio-demographic and obstetric characteristics of pregnant women (n = 228)

Variable	N (%)	Variable	N (%)
**Age, (years), Mean (SD)**	29.0 (5.9)	**Planned pregnancy**	
**Husband age, (years), Mean (SD)**	33.9 (5.7)	Yes	159 (69.7)
**Education**		No	69 (30.3)
Under diploma	80 (35.1)	**Wanted sex of baby**	
Diploma	91 (39.1)	Yes	180 (78.9)
Academic	57 (25.0)	No	48 (21.1)
**Husband education**		**Satisfaction with pervious birth**	
Under diploma	86 (37.7)	Yes	78 (67.8)
Diploma	87 (38.2)	No	37 (32.2)
Academic	55 (24.1)	**Complications during pregnancy** ^**a**^	
**Job**		Yes	53 (23.2)
Housewife	215 (94.3)	No	175 (76.8)
Employed	13 (5.7)	**Type of birth**	
**Husband job**		Vaginal	99 (43.4)
Employed	37 (16.2)	Emergency cesarean section	55 (24.1)
Worker	48 (21.1)	Elective cesarean section	74 (32.5)
Self-employed	143 (62.7)	**Place of birth**	
**Income satisfaction**		Teaching	63 (28.1)
Not enough	51 (22.4)	Organizational	65 (29.0)
Relatively enough	154 (67.5)	Private	96 (42.9)
Completely enough	23 (10.1)	**Postpartum complication** ^**b**^	
**Gestational age at entering in the study (Weeks), Mean (SD)**	31.9 (2.7)	Yes	29 (12.7)
**BMI (kg/m2), Mean (SD)**	24.9 (4.4)	No	199 (87.3)
**Parity**		**Skin to skin contact**	
1	99 (43.4)	Yes	190 (84.8)
2	84 (36.8)	No	34 (15.2)
3	45 (19.7)	**Baby weight (gr), Mean (SD)**	3119.4 (530.2)
**History of abortion**		**Baby height (cm), Mean (SD)**	50.5 (3.0)
Yes	41 (18.0)	**Baby head (cm), Mean (SD)**	34.0 (1.8)
No	187 (82.0)	**Baby sex**	
**History of stillbirth**		Boy	122 (53.5)
Yes	3 (1.3)	Girl	102 (44.7)
No	225 (98.7)		
**Wanted pregnancy**			
Yes	167 (73.2)		
No	61 (26.8)		

^a^ Preeclampsia, Diabetes mellitus; ^b^ Infection, Hemorrhage, Thrombosis

Based on the results of the Pearson’s correlation test, there was no statistically significant relationship between feelings of being hassled during pregnancy and MFA. (r= -0.03, p = 0.597). However, there was a statistically significant relationship between feelings of being uplifted during pregnancy and MFA (r = 0.50, p < 0.001) (Table 2). According to the results of the Spearman’s test, there was no statistically significant relationship between pregnancy experiences (hassles and uplifts) (r = 0.01, p = 0.887) and postpartum bonding (r= -0.03, p = 0.603). Also, there was no statistically significant relationship between childbirth experience and postpartum bonding (r= -0.13, p = 0.100) and between MFA and postpartum bonding (r= -0.05, p = 0.425) (Table 2).

**Table 2:** Relationship of pregnancy experience with maternal-fetal attachment and postpartum bonding (n = 228) ^*^

Variables	Mean (SD)		Uplifts	Hassles	Childbirth Experience	Postpartum Bonding
			r (p-value) ^**^			
**Postpartum Bonding (Range = 0- 125)**	19.5 (7.6)	-0.03 (0.603)		0.01 (0.887)	-0.13 (0.100)	-
**Maternal-Fetal Attachment (24–120)**	91 (10.3)	0.50 (< 0.001)		-0.03 (0.597)	-	-0.05 (0.425)
**Pregnancy Experience**		-		-	-	-
**Uplifts (0–30)**	22. 2 (4.5)	-		-	-	-
**Hassles (0–30)**	11.1 (5.2)	-		-	-	-
**Childbirth Experience (1–4)**	2.8 (0.4)	-		-	-	-

* Sample size for postpartum bonding and childbirth experience was 224 women; ** Pearson Correlation test was done except for postpartum bonding

Based on the results of univariate tests, there was statistically a significant relationship between the variables of spouse’s age, gestational age, parity, wanted and planned pregnancy, and wanted sex of baby with MFA (p < 0.05). Also, there was a statistically significant relationship between the variables of income status, gestational age, and kind of birth with postpartum bonding (p < 0.05) (Table 3).

**Table 3:** Relationship of socio-demographic and obstetric characteristics with maternal-fetal attachment and postpartum bonding (n = 228)

Variable	Maternal-Fetal Attachment (n = 228)		Postpartum Bonding (n = 224)	
	Mean (SD)	P	Mean (SD)	p
**Age (years), Mean (SD)**	0.08	0.230	0.01	0.843
**Husband age (years), Mean (SD)**	0.13	0.036	0.04	0.535
**Education**		0.118		0.282
Under diploma	89.78 (11.64)		18.44 (8.88)	
Diploma	92.49 (9.27)		19.92 (7.47)	
Academic	91.15 (9.91)		20.42 (7.95)	
**Husband Education**		0.408		0.076
Under diploma	89.87 (11.93)		19.11 (8.54)	
Diploma	91.43 (10.01)		18.64 (7.24)	
Academic	92.10 (8.05)		21.54 (6.68)	
**Job**		0.765		0.825
Housewife	90.95 (10.44)		19.56 (7.79)	
Employed	91.48 (9.34)		19.07 (6.04)	
**Husband job**		0.287		0.627
Employed	92.91 (8.66)		20.10 (7.02)	
Worker	91.93 (12.75)		18.62 (8.87)	
Self-employed	90.20 (9.85)		19.69 (7.45)	
**Income satisfaction**		0.129		0.001
Not enough	55.56 (10.51)		23.07 (6.74)	
Relatively enough	52.19 (10.40)		18.68 (7.71)	
Completely enough	52.60 (9.25)		17.21 (7.32)	
**Gestational age (Weeks), Mean (SD)**	-0.16	0.013	-0.18	0.006
**BMI (kg/m** ^**2**^ **), Mean (SD)**	0.05	0.435	-	-
**Parity**		0.018		0.831
1	92.41 (9.58)		19.86 (7.80)	
2	91.11 (1.04)		19.39 (7.42)	
3 or more	87.33 (11.85)		19.06 (8.08)	
**History of abortion**		0.331		0.576
Yes	92.43 (11.73)		18.92 (7.68)	
No	90.69 (10.05)		19.67 (7.71)	
**History of stillbirth**		0.739		0.064
Yes	93.00 (13.74)		9.50 (0.70)	
No	90.98 (10.35)		19.62 (7.66)	
**Wanted pregnancy**		0.024		0.270
Yes	91.94 (10.18)		19.88 (8.00)	
No	88.44 (10.51)		18.60 (6.77)	
**Planned pregnancy**		0.003		0.221
Yes	92.35 (10.12)		19.94 (6.60)	
No	87.91 (10.35)		19.94 (8.09)	
**Wanted sex of baby**		0.002		0.720
Yes	92.10 (10.78)		19.44 (7.90)	
No	86.91 (7.42)		19.90 (6.83)	
**Satisfaction with pervious birth**		0.172		0.774
Yes	88.00 (9.41)		19.12 (8.21)	
No	91.00 (13.62)		19.56 (6.70)	
**Complications during pregnancy** ^**a**^		0.689		0.067
Yes	91.50 (9.20)		21.25 (8.90)	
No	90.85 (10.72)		19.01 (7.23)	
**Type of birth**		-		0.029
Vaginal			18.08 (7.72)	
Emergency cesarean section			19.94 (6.60)	
Elective cesarean section			21.20 (8.12)	
**Postpartum complication** ^**b**^		-		0.402
Yes			20.65 (7.99)	
No			19.36 (7.65)	
**Skin to skin contact**		-		0.382
Yes			19.72 (7.76)	
No			18.47 (7.27)	
**Baby sex**		-		0.360
Boy			19.96 (8.43)	
Girl			19.01 (6.70)	
**Baby weight**		-	0.00	0.907

^a^ Preeclampsia, Diabetes mellitus; ^b^ Infection, Hemorrhage, Thrombosis

After adjusting for possible confounding variables, the mean score of MFA was significantly higher among women with feelings of being uplifted during pregnancy [β (95% CI) = 1.14 (0.87 to 1.41); p < 0.001]. MFA score was lower in women with second birth compared to those with third birth [β= -3.41 (-6.81 to -0.02); p = 0.049]. MFA score was lower in women with unwanted sex of fetus compared to those with wanted sex of fetus [β= -3.13 (-6.19 to -0.07); p = 0.045] (Table 4).

**Table 4:** Relationship of maternal-fetal attachment with pregnancy and birth experience (n = 228)

Variable	Maternal-Fetal Attachment	
	β (95% CI)	P^*^
**Uplifts**	1.14 (0.87 to 1.41)	< 0.001
**Hassles**	0.04 (-0.17 to 0.26)	0.691
**Husband age (Years), Mean (SD)**	-0.08 (-0.31 to 0.14)	0.476
**Gestational age (Weeks), Mean (SD)**	0.74 (0.32 to 1.15)	< 0.001
**Parity (Reference: 3**)		
1	0.97 (-2.74 to 4.68)	0.607
2	-3.41 (-6.81 to -0.02)	0.049
**Wanted pregnancy (Ref: Yes)**		
No	0.17 (-6.47 to 6.81)	0.959
**Planned pregnancy (Ref: Yes)**		
No	-1.06 (-7.67 to 5.55)	0.752
**Wanted sex of baby (Ref: Yes)**		
No	-3.13 (-6.19 to -0.07)	0.045
**R** ^**2**^	0.329	

*General Linear Model

After adjusting for potential confounding variables, we witnessed that women who reported insufficient household income for covering living expenses had a more unfavorable postpartum bonding compared to those who were satisfied with their income [β = 5.61 (1.13 to 10.09); p = 0.014] (Table 5).

**Table 5:** Relationship of mother-child bonding with pregnancy and birth experience (n = 228)

Variable	Postpartum Bonding	
	β (95% CI)	P^*^
**Uplifts**	-0.096 (-0.39 to 0.20)	0.533
**Hassles**	-0.06 (-0.30 to 0.16)	0.573
**Childbirth experience**	-1.24 (-4.27 to 1.79)	0.419
**Gestational age (Weeks), Mean (SD)**	-0.39 (-0.84 to 0.05)	0.085
**Income satisfaction** **(Ref: Completely enough)**		
Not enough	5.61 (1.13 to 10.09)	0.014
Relatively enough	-0.04 (-3.83 to 3.74)	0.981
**Complication during pregnancy (Ref: Yes)**		
No	-1.78 (-4.65 to 1.09)	0.223
**Type of birth (Ref: C-section)**		
Vaginal	-1.02 (-3.45 to 1.39)	0.404
**R** ^**2**^	0.179	

*General Linear Model

## Discussion

The present study was conducted with the aim of determining the relationship between pregnancy and birth experience with MFA and postpartum bonding.

According to our results, while there was a direct statistical relationship between feelings of being uplifted during pregnancy and MFA, there was no relationship between feelings of being hassled and MFA. In confirmation of this finding, the results of a longitudinal study in the United States on 158 pregnant women showed a positive relationship between pregnancy uplifts and MFA. However, there was no significant relationship between hassles and MFA [25]. In contrast, the results of a study on 186 pregnant women in Colombia reported a negative association between pregnancy experience, especially hassles, and MFA [26]. Also, the findings of a study in Denmark reported a reverse relationship between pregnancy experience and MFA; in this study, there were women from African-American, Latin, and Egyptian races, but not Asians [27]. Other studies reported that negative emotions during pregnancy were related to poor maternal-infant attachment. In these studies, depression and anxiety scales were used to measure the presence of negative emotions in mothers [5, 28]. However, in our study, we used the PEQ to measure the negative feelings experienced during pregnancy, which could be a potential factor for the inconsistency between this study and the above-mentioned studies. The presence of women from diverse racial backgrounds, as well as difference in inclusion and exclusion criteria and sample size could also account for the observed inconsistencies.

In present study, there was no significant relationship between pregnancy experience and postpartum bonding. One study investigated the relationship between anxiety disorders in pregnancy and the quality of mother-baby bonding among 454 pregnant women in London. The results indicated that pregnancy anxiety was associated with higher perceived bonding disorder; however, there was no association between pregnancy anxiety and poor quality of mother-infant interaction. The study also revealed that higher levels of depressive symptoms were associated with lower maternal sensitivity [29]. Also, a longitudinal study in the United States reported a significant relationship between woman’s emotional appraisal of pregnancy and postpartum bonding [25]. Another longitudinal study conducted in Italy reported a significant inverse relationship between stressful life events experienced during pregnancy and postpartum bonding [30]. The inconsistency in findings may be attributed to differences in scales used and inclusion criteria employed in the studies. Also, it is possible that mother-child bonding is more influenced by postpartum events, such as postpartum depression, anxiety, and social support, rather than events occurring during pregnancy.

There was no statistically significant relationship between childbirth experience and postpartum bonding in our study. This finding was inconsistent with a longitudinal prospective cohort study at the University of Dresden, Germany, which reported a positive childbirth experience had an effect on the strengthening of mother-child bonding [12]. According to a Missouri review, mothers who were stressed during labour had unsatisfactory communication with their babies after birth [31]. In a prospective cohort study in Germany, the mother’s negative experiences during childbirth (such as fear of childbirth, stress, and anxiety) led to creating postpartum bonding disorders [32]. The probable reasons for the inconsistency of the results can be due to the following items: different scales, racial differences, differences in demographic and obstetrics characteristics, sample size, and the measurement of depression and anxiety in pregnancy. In the present study, the occurrence of cesarean birth was unavoidable due to the study design and inclusion of women during pregnancy. The type of birth has been identified as one of the potential factors that can influence postpartum bonding [33]. In our study, there was a significant relationship between the type of birth and postpartum bonding. Although the effect of birth type on bonding was adjusted using multivariate analysis, it can still affect the results of this study as a confounding factor.

In our study, there was no statistically significant relationship between MFA and postpartum bonding. The results of a review study indicated a weak relationship between MFA and mother-child bonding [5]. A prospective cohort study in Italy reported a positive significant relationship between maternal attachment and mother-child bonding [34]. A longitudinal study in Italy reported a direct relationship between MFA (with the mean attachment score of 78.01) during pregnancy with mother-child bonding up to four months following birth [35]. Thus, other variables may be considered as effective mediating factors in the relationship between attachment and bonding.

In the present study, the mean score of MFA was significantly higher among women with higher gestational age. In confirming this finding, a cross-sectional study in Italy showed a significant direct relationship between gestational age and MFA [36]. In a longitudinal study in the United States, maternal-infant attachment score increased with increasing gestational age [25]. The results of this study showed multiparity as a factor to reduce MFA. Consistent with the finding of present study, two studies conducted in India [37] and Turkey [38] also reported a statistically significant association between MFA and parity. In a descriptive-comparative study in Iran, the attachment score decreased with increasing parity [39].

In the current study, wanted sex of baby was associated with higher score of MFA. According to the findings of a descriptive study in Egypt [40], the favorability of the gender of the fetus was reported to be an effective factor in strengthening the mother’s attachment. Also, in a study in turkey [41] on mother-fetus attachment, wanted sex of baby was introduced as one of the predictive factors. In the present study, insufficient income level was associated with poor mother-child attachment. Findings from a cross-sectional study in Slovenia showed that women with low income were at risk of mental health problems; so, low-income mothers were more likely to neglect their child’s emotional needs [42].

The main strengths of the study included, using standard scales to measure the variables, and controlling demographic confounders. Also, to adjust the effect of the confounders, multivariate general linear model was used. Nonetheless, the limitations of the study include the following: lack of assessment of anxiety and depression during pregnancy; participation in the prenatal class; a wide range of gestational age (8-week) at the time of recruiting women in the study, which can affect MFA and pregnancy experience; and not measuring social support. Considering the limited number of studies in this field, it is recommended to conduct further studies and provide prenatal counseling by health personnel to make pregnancy pleasant.

## Conclusion

The results of this study showed the positive relationship between feelings of being uplifted during pregnancy and MFA. Hence, implementation of intervention programs according to the mother’s needs to increase happiness and peace may have a positive effect on MFA.

## Data Availability

The datasets generated and/or analyzed during the current study are not publicly available due to the limitations of ethical approval involving the patient data and anonymity but are available from the corresponding author upon reasonable requests.

## Abbreviations

PES: Pregnancy Experience Questionnaire
MFAQ: Maternal-Fetal Attachment Questionnaire
CEQ 2.0: Childbirth Experience 2.0
PBQ: Postpartum Bonding Questionnaire
